# Design of Al-decorated C_24_N_24_ fullerene for efficient adsorption and removal of methylene blue dye from water

**DOI:** 10.1039/d6ra00172f

**Published:** 2026-02-19

**Authors:** Habib Ullah, Zakir Zaman Khan, Akif Safeen, Adnan Ali Khan, Noor Ul Islam, Ghafar Ali, Basit Ali, Imran Shakir, Yi Xie

**Affiliations:** a State Key Laboratory of Silicate Materials for Architectures, Wuhan University of Technology Wuhan 430070 P.R. China xiey@whut.edu.cn; b Department of Chemistry, University of Malakand Dir Lower Chakdara 18800, Khyber Pakhtunkhwa Pakistan; c Department of Physics, University of Poonch Rawalakot Rawalakot 12350 Pakistan; d School of Science, Harbin Institute of Technology (Shenzhen) Shenzhen China; e Department of Chemistry, Government Degree College Lalqilla Dir Lower Lalqilla 18350 Khyber Pakhtunkhwa Pakistan; f Nanomaterials Research Group (NRG), Physics Division PINSTECH, Nilore Islamabad Pakistan; g Department of Chemistry and Materials Science, School of Chemical Engineering, Aalto University P.O. Box 16100 FI-00076 Aalto Finland basit.ali@aalto.fi; h Department of Physics, Faculty of Science, Islamic University of Madinah Madinah 42351 Saudi Arabia

## Abstract

In this study, density functional theory (DFT) is used to explore aluminum-doped porphyrin-like porous fullerene (Al_6_@C_24_N_24_) as a potential adsorbent for scavenging aquatic carcinogenic methylene blue (MB) dye. The Al_6_@C_24_N_24_ system demonstrates thermal stability up to 1000 K, implying the robust incorporation of Al atoms into the C_24_N_24_ framework. The adsorption analysis at the DFT-D3 level reveals that MB dye is efficiently adsorbed on the surface of the Al_6_@C_24_N_24_ framework, with adsorption energies ranging from −2.03 to −2.97 eV. The charge-density-difference (CDD) mapping, partial-density of states (PDOS), and quantum theory of atoms-in-molecules (QTAIM) analyses validate the electrostatic interactions, facilitating MB chemisorption on the Al_6_@C_24_N_24_ surface. The maximum uptake capacity assessment indicates that the Al_6_@C_24_N_24_ system can effectively adsorb up to six MB molecules, highlighting its potential for efficient dye scavenging. Moreover, molecular dynamics (MD) simulations demonstrate the thermodynamically feasible formation of the 6MB-Al_6_@C_24_N_24_ complex at 300 K in an aqueous environment, substantiating the existence of the complex in real scenarios. These findings provide a theoretical basis for experimental investigations, suggesting that Al_6_@C_24_N_24_ could serve as an innovative wastewater purifier by scavenging organic carcinogenic dyes, contributing to advancements in environmental remediation technologies.

## Abstract 2

In this study, aluminum-doped porphyrin-like porous fullerene (Al@C24N24) is used as a potential adsorbent for scavenging aquatic carcinogenic methylene blue (MB) dye.

## Graphical abstract

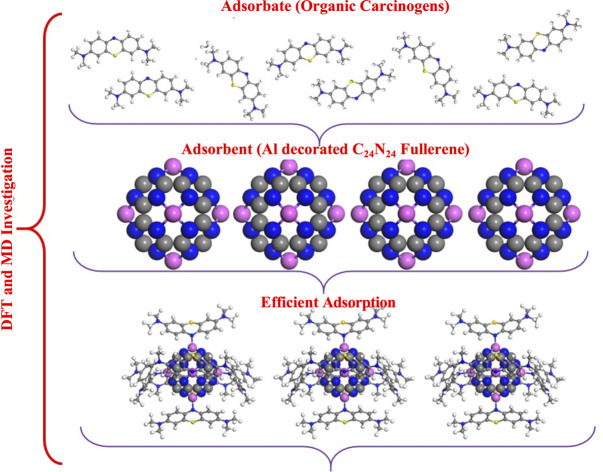

## 1. Introduction

Methylene blue (MB) is a water-soluble phenothiazinium dye with a stable structure and a dark green appearance^1–3^ that is commonly used for a spectrum of applications, both industrial and non-industrial, as a dyeing and colouring additive.^4–10^ Apart from its applications, MB can cause adverse effects on human health, including neurotoxicity, hemolytic anemia, carcinogenicity, mutagenicity, cardiovascular, and reproductive effects, along with gastrointestinal and discoloration issues.^11–14^ Due to its extensive use, MB often enters the natural aquatic ecosystem, posing a risk not only to human health but also to the overall environment.^3,15^

To address the challenges of removing such carcinogenic pollutants, several treatment methods, including membrane filtration and advanced oxidation processes, have been reported.^1–3,10^ The effectiveness of these methods largely depends on the physicochemical characteristics of the micro-pollutants and specific operational parameters. Optimizing these variables is crucial for achieving efficient pollutant removal under diverse environmental conditions.^3^ On the other hand, adsorption presents a straightforward and effective method for scavenging hazardous pollutants, including MB from wastewater.^16^ Due to its uncomplicated design, low cost, resilience to various toxic substances, and operational simplicity, adsorption is often favoured over other wastewater treatment techniques.^17^

Numerous nanomaterials, such as graphene, graphene oxide, activated carbon, and various metals and metal oxides, have been investigated as adsorbents for the effective removal of dyes from wastewater, both experimentally and computationally.^18–22^ Specifically, MB dye has been removed successfully from wastewater using activated carbon in experimental setups,^1,2,4,10,15,21,23^ whereas metal oxides and other nanomaterial adsorbents have been reported in computational studies.^23–26^ Despite these advancements, there remains a significant need to explore novel adsorbents that combine high porosity, excellent adsorption capacity, environmental safety, and cost-effectiveness. Moreover, gaining a deeper understanding of the adsorption mechanisms at the molecular level is essential for optimizing dye-removal processes.

In recent years, nitrogen-doped porous carbon-based nanomaterials have attracted considerable attention as emerging adsorbents due to their economical synthesis methods, large surface areas, and remarkable adsorption properties.^27–31^ Considering the synthesis of these nanomaterials, reactive-magnetron sputtering and chemical-vapor deposition (CVD) are the most common methods for their fabrication.^32–34^ Introducing nitrogen atoms into carbon-based structures, such as fullerenes, can alter the carbon π-electron system as a result of charge transfer between the nitrogen and carbon atoms. Consequently, the polar C–N bonds in nitrogen-doped carbon frameworks become preferred sites for nucleophilic or electrophilic interactions.^35^ A novel form of porous fullerene, represented as C_24_N_24_, comprising eight triazine rings connected by C–C bonds, was theoretically proposed by Srinivasu and Ghosh, highlighting its potential as an advanced adsorbent material.^36^ Furthermore, this innovative porous nanocluster (fullerene) is characterized by nitrogen-containing cavities similar in structure to those found in porphyrin molecules, with six such nitrogen sites present in C_24_N_24_ fullerene.^36^ Studies have shown that when C_24_N_24_ fullerenes are doped with metals, such as lithium, aluminium, and various transition metals, they can serve as highly effective catalysts and hydrogen-storage materials.^36–38^ Specifically, C_24_N_24_ fullerenes decorated with iron or silicon atoms have displayed remarkable catalytic performance, notably facilitating nitrogen oxide (NO) reduction and carbon monoxide (CO) oxidation reactions.^39–41^

Research indicates that N_4_ cavities in C_24_N_24_ fullerenes serve as optimal sites for binding foreign atoms, such as metals and metalloids, through robust N–X covalent bonds (where *X* represents the metal or metalloid). This bonding minimizes the risk of atom clustering or aggregation on the fullerene surface, maintaining a stable distribution. In computational studies, achieving high chemical accuracy in predicting structural and electronic properties requires careful consideration of several factors, for instance, a large basis set, incorporation of relativistic effects, and accurate treatment of electronic correlations. When these conditions are fulfilled, simulations can yield precise insights into the material's properties.^42^

The present work investigates the adsorption of methylene blue (MB) dye on aluminium-decorated C_24_N_24_ fullerene (Al_6_@C_24_N_24_), using first-principles simulations. The incorporation of an Al atom into the N_4_ cavity of C_24_N_24_ significantly enhances its interaction with MB dye molecules. The observed high negative adsorption energies for MB molecules over the Al active sites on the surface of the Al_6_@C_24_N_24_ adsorbent suggest their robust interaction. These computational findings indicate that Al_6_@C_24_N_24_ holds great promise as an adsorbent material for scavenging toxic dyes from aqueous environments.

## 2. Computational details

In this study, spin-polarized density functional theory (DFT) implemented in the DMol^3^ package was adopted for the overall simulations.^43,44^ Structural optimizations and electronic property evaluations were carried out using the Perdew–Burke–Ernzerhof (PBE) exchange-correlation functional under the generalized-gradient approximation (GGA) framework,^45^ along with the double-numerical-plus-polarization (DNP) basis set.^46^ To ensure stability of the optimized structures, Hessian analyses were conducted at the same theoretical level and basis set, whereas the absence of negative vibrations confirmed structural stability. Key computational parameters included the basis set cutoff of 4.6 Å in addition to the thermal-smearing of 0.136 eV. Moreover, Grimme's DFT-D3 empirical method was applied to consider the van der Waals interactions.^47,48^ Structural relaxations were performed with convergence thresholds of 0.027 eV Å^−1^ for force, 0.005 Å for maximum displacement, and 2.72 × 10^−4^ eV for energy.^49^ Additionally, the implicit solvation effect was introduced using the conductor-like screening model (COSMO) with water as the solvent (*ε* = 78.5) to study the effect of solvation in an aqueous medium. The energy of adsorption (*E*_ad_) of different complex structures was determined as:

1 *E*_ad_ = *E*_complex_−(*E*_adsorbent_ + *E*_adsorbate_)

where the terms *E*_complex_, *E*_adsorbent_, and *E*_adsorbate_ refer to the ground-state optimized energies of MB-adsorbed Al@C_24_N_24_ (complex), Al-doped C_24_N_24_ (adsorbent), and the MB molecule (adsorbate), respectively. Additionally, the *E*_ad_ of Al atom incorporation into C_24_N_24_ was calculated to evaluate the stability of the Al-coordinated structure:

2 *E*_ad_ = *E*_Al@C_24_N_24__−(*E*_C_24_N_24__+*E*_Al_)

Here, *E*_Al@C_24_N_24__ and *E*_C_24_N_24__ are the ground-state optimized energies of Al-incorporated and bare C_24_N_24_ nano-architecture, respectively, whereas *E*_Al_ denotes the isolated Al atom energy.

## 3. Results and discussion

### 3.1 Geometry, electronic properties, and stability of Al@C_24_N_24_

In our initial investigations, we examined the structural and electronic characteristics of both the bare and Al-decorated C_24_N_24_ nanocages, along with the MB dye molecule. The optimized geometries and electronic properties of the bare C_24_N_24_ nanocage and MB dye are presented in Fig. S1. As illustrated, the C_24_N_24_ nanocage (Fig. S1a) features highly electronegative N_4_ cavities, resembling those in porphyrin structures, which have been identified as ideal sites for metal or metalloid anchoring in previous studies.^50–54^ This fullerene comprises eight triazine rings that are interconnected by C–C bonds. Calculations indicate that the C–N and C–C bond distances are approximately 1.34 and 1.55 Å, respectively, which are consistent with the previous findings.^50,51,53,54^ Additionally, the MEP map (Fig. S1c) elaborates the electronegative nature of the N_4_ cavities, where the four electron-rich nitrogen atoms are shown by red spots (like corners) in the cavity. Earlier research has further confirmed that the N_4_ cavity within the C_24_N_24_ framework is a preferred anchoring site for foreign atoms, such as silicon and aluminium.^54,55^ Therefore, in this study, an aluminum atom was initially positioned within the N_4_ cavity of a C_24_N_24_ nanocage, followed by full geometric relaxation.

**Fig. 1:**
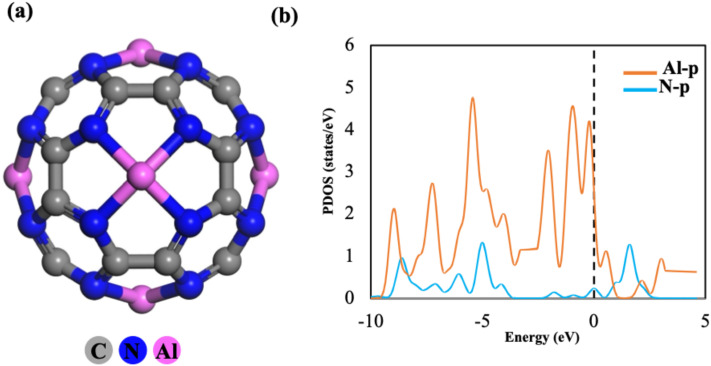
Optimized geometry of six Al-incorporated C_24_N_24_ fullerene (Al_6_@C_24_N_24_) (a) and its corresponding partial density of states plot (b).

Optimized configurations of C_24_N_24_ fullerenes incorporating a single Al atom and six Al atoms (Al@C_24_N_24_ and Al_6_@C_24_N_24_), along with their corresponding partial density of states (PDOS) plots, can be viewed in Fig. 1 and S2 respectively. These post-optimized geometries revealed that the Al atom acquires the top-center position of the N_4_ cavity plane, forming four equivalent Al–N bonds with nearby nitrogen atoms, each measuring approximately 1.87 Å. This bond length is marginally longer than that observed in Si@C_24_N_24_ (1.82 Å) and aligns closely with the previously reported value (1.87 Å) for Al@C_24_N_24_.^55,56^ The N–Al–N bond angles are around 147.6°, indicating a slight deviation of the Al atom from the N_4_ plane. The calculated *E*_ad_ value of Al on C_24_N_24_ is approximately −6.91 eV at the DFT-D3 level, indicating a strong interaction between the Al and the neighbouring nitrogen atoms, which would potentially inhibit the diffusion and clustering of Al atoms within the nanocage.^50,51,55,56^ Similarly, Hirshfeld charge-transfer analysis suggests that almost 0.50 e is withdrawn by the adjacent nitrogen atoms from the centrosymmetric Al atom, conferring ionic character to the Al–N bond by the notably uneven charge distribution. Additionally, the PDOS analysis (Fig. S2c) reveals strong hybridization between the p-orbitals of the Al and N atoms at the Fermi level, further supporting the robust Al–N bond formation within the C_24_N_24_ structure.^57–59^

As indicated by the optimized geometry of pristine C_24_N_24_ fullerene (Fig. S1a), each N_4_ cavity can serve as an excellent site for Al adsorption, as corroborated in reported studies.^56^ Accordingly, this study explores the adsorption of Al atoms across all the N_4_ cavities. The optimized geometry of the Al_6_@C_24_N_24_ complex (Fig. 1) demonstrates that each Al atom is firmly captured within an N_4_ cavity, adopting a tetragonal-pyramidal coordination with neighbouring nitrogen atoms, consistent with previous findings.^50,51,55,56^ The Al–N bond lengths were calculated to be around 1.91 Å, which are slightly longer than those observed in single Al@C_24_N_24_. The average *E*_ad_ value of each Al atom was approximately −5.18 eV, suggesting a gradual decrease in affinity of the nanocage for Al adsorption as the number of adsorbed Al atoms increases. Moreover, the PDOS analysis (Fig. 1b) reveals an overlap between the p-orbitals of Al and N atoms below the Fermi level, indicating a slight weakening of the Al–N bonds with each additional Al atom. Furthermore, Hirshfeld charge analysis showed a decrease in the atomic charge of Al atoms to 0.54 e upon multiple Al adsorption in the fullerene, leading to an enhancement in the Lewis-acidic characteristics and subsequent surface reactivity of the Al@C_24_N_24_ nanomaterial.

Previous literature^60–62^ highlighted the significant influence of surface-charge density distribution on the adsorption behaviour of MB molecules on nanomaterials. In this context, the MEP maps of Al_6_@C_24_N_24_ and Al@C_24_N_24_ complexes are illustrated in Fig. S3a and S4b, respectively. As depicted in blue, the electron-depleted regions are prominent around the Al atoms, indicating the electropositive nature of Al atoms. Given the higher electronegativity difference between N and Al (3.04 *vs.* 1.61, based on the Pauling scale), the N atoms exhibit a more nucleophilic character in comparison to the electrophilic nature of the Al atom within the Al_6_@C_24_N_24_ nanostructure. Likewise, the charge-density difference (CDD) (Fig. S3b) and deformation-charge density (DCD) (Fig. S3c) analyses support these findings, showing a notable electron density reduction on the Al atom, further affirming its electropositive character. This suggests that Al atoms are capable of forming electrostatic interactions with the negatively charged fullerene adsorbate.

The quantum theory of atoms-in-molecules (QTAIM) analysis is usually performed to characterize the nature of a chemical bond through AIM parameters, such as the electron density *ρ*(*r*), the Laplacian of electron density ∇^2^*ρ*(*r*), and the total energy density *H*(*r*). According to QTAIM theory, if ∇^2^*ρ*(*r*) < 0 and *H*(*r*) < 0 at a given bond critical point (BCP), the bond would be a shared shell (covalent) interaction. However, if both ∇^2^*ρ*(*r*) and *H*(r) are positive (>0), the interaction would be a closed shell (purely electrostatic, noncovalent or weak H-bond) interaction. Alternatively, the interaction would be a polar covalent or a strong H-bond if ∇^2^*ρ*(*r*) > 0 and *H*(r) < 0.^63,64^ The molecular graphs of Al_6_@C24N_24_ and Al@C_24_N_24_ complexes are displayed in Fig. S3d and S5, respectively, whereas the QTAIM parameters are listed in Table S1. According to the above criteria, the Al–N bonding within the C_24_N_24_ framework is primarily governed by polar covalent interactions, since ∇^2^*ρ*(*r*) values are positive and *H*(*r*) values are negative. An analogous idea regarding the electrostatic nature of the Al–N bond is provided by the CDD and DCD maps in Fig. S3b and c), which further infer the stability and integrity of the Al_6_@C_24_N_24_ complex. In addition, the HOMO–LUMO energy gap for Al_6_@C_24_N_24_ was calculated to be 0.97 eV (Fig. S3e and f), highlighting the electronic characteristics of the system.

To evaluate the thermal stability of the Al_6_@C_24_N_24_ system, *ab initio* molecular dynamics (AIMD) simulations were conducted. These simulations were performed at temperatures of 500 and 1000 K over a period of 2 ps and 10 ps. The final geometries obtained from the AIMD simulations are presented in Fig. 7a and b and S6. Remarkably, Al_6_@C_24_N_24_ maintained its structural integrity at both temperatures, with all the Al atoms maintaining their positions above the N_4_ cavities. Yet, minor fluctuations in the Al–N bond distances were observed, suggesting the strong polar covalent nature of the bond between the Al and N atoms. Based on these findings, we propose that the Al_6_@C_24_N_24_ system exhibits high thermal stability, remaining structurally resilient at temperatures up to 1000 K.

### 3.2 Application of Al@C_24_N_24_ as an adsorbent for MB removal

To explore the MB dye adsorption efficiency of the Al@C_24_N_24_ complex as an adsorbent for scavenging carcinogenic dyes from water, we examined the adsorption characteristics of the MB dye on the original C_24_N_24_ surface, as investigated earlier.^65^ The optimized structure of the MB@C_24_N_24_ complex, along with its corresponding CDD and PDOS plots, is shown in Fig. S8a and c. Key parameters, including bond lengths, *E*_ad_ value, and charge transfer, are presented in Table S3, which are in agreement with previously reported data.^65^ From the geometry relaxation analysis, it is observed that the MB molecule binds to the surface of bare C_24_N_24_ fullerene *via* two H⋯N intermolecular bonds, measuring between 2.38 and 2.42 Å, indicating a physisorption interaction. The calculated *E*_ad_ value of this complex is −0.49 eV. Hirshfeld charge-transfer analysis reveals a minimal uptake of charges by hydrogen atoms of the MB from the C_24_N_24_ surface. This idea is further verified by CDD analysis, as depicted in Fig. S8b, which shows no significant electron-density overlap between the N and H atoms of the C_24_N_24_ nanocage and the MB dye, respectively. Analogously, PDOS analysis (Fig. S8c) further supports the above-mentioned findings; here, the absence of electronic-states coupling around the Fermi level between the C_24_N_24_ fullerene and MB signifies a physical interaction rather than chemical bonding. To further verify these physical interactions, we conducted AIM analysis. Fig. S9 displays the molecular graph, while Table S3 summarizes the AIM parameters. The very small values of *ρ*(*r*) (0.008 a.u.) and the positive values of ∇^2^*ρ*(*r*) and *H*(*r*), reveal the existence of weak hydrogen bonds (H⋯N) and van der Waals forces between the MB dye and the C_24_N_24_ surface,^61,62^ indicating the dye molecule is only loosely adsorbed onto the pristine C_24_N_24_ system.

**Fig. 2:**
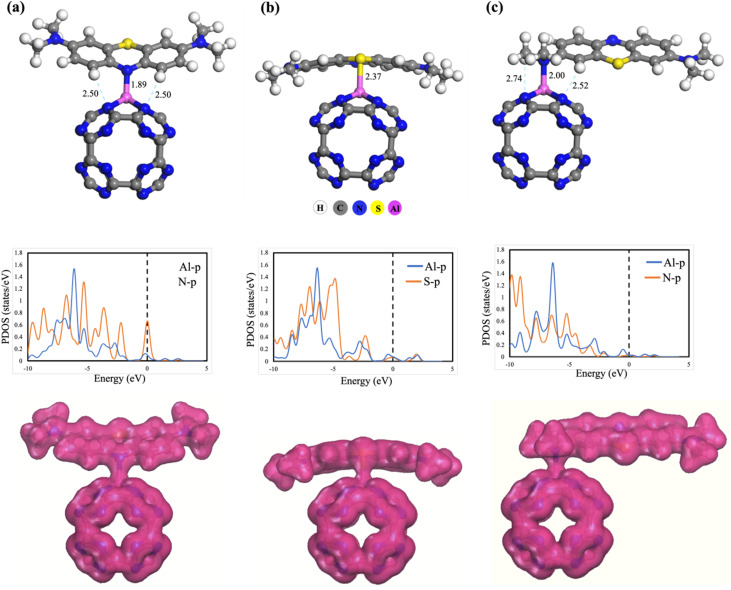
The optimized geometries of Al@C_24_N_24_ complexes with MB adsorption *via* the N(1) site (a), the S site (b), and the N(2) site (c), and their corresponding partial density of states and charge density difference plot. All distances are in Å.

To enhance the adsorption capability of the C_24_N_24_ system for scavenging MB dye from water, the system is modified by incorporating Al atoms. Fig. 2 illustrates the different possible interactions of MB dye with the Al-decorated C_24_N_24_ fullerene. Based on the MEP map of the MB (Fig. S1d), the nitrogen (N1) atom, present in the central ring, is recognised as the most nucleophilic site, carrying −0.185 e charge density. Consequently, the N1 atom of MB is chosen for the interaction with the Al atom of the Al@C_24_N_24_ system. Furthermore, sulphur (S) in the central ring and the nitrogen (N2) atom in the H_3_C–N–CH_3_ moieties at the edges of the MB molecule are also considered for the interaction with the Al atom. In the first adsorption configuration (configuration-1), given in Fig. 2a, the MB dye is positioned in such a way that the N1 atom faces the Al atom of the Al@C_24_N_24_ system. After structural optimization, it is observed that the N1 atom forms a strong covalent bond with the Al atom, with a bond distance of 1.89 Å. Likewise, intermolecular hydrogen bonding (H⋯N) occurs between the dye and the N atoms of the Al@C_24_N_24_ system, with a bond distance of 2.50 Å. These interactions result in a high *E*_ad_ value of −2.97 eV at the DFT-D3 level, as summarized in Table 1. Hirshfeld charge analysis reveals a reduction in the positive charge of the Al atom from 0.507 to 0.399 e, indicating a charge transfer of −0.109 e from MB to the adsorbent. In the second adsorption configuration (configuration-2), shown in Fig. 2b, the MB molecule is aligned above the Al@C_24_N_24_ in such a way that the S atom of the central ring of MB interacts with the Al atom of the adsorbent. After structural relaxation, a S–Al covalent bond, with a length of 2.37 Å, is observed, which is deemed responsible for the adsorption of MB over the fullerene surface at the cost of −2.08 eV as *E*_ad_, as shown in Table 1. Hirshfeld analysis indicates a charge transfer of −0.149 e from the S atom of the dye to the Al atom. In the third adsorption configuration (configuration-3), presented in Fig. 2c, the MB dye interacts *via* the N2 atom of the H_3_C–N–CH_3_ group. Geometry optimization shows the formation of an Al–N bond with a bond distance of 2.0 Å. Additionally, two weak hydrogen bonds are observed, with bond lengths of 2.74 Å and 2.52 Å. The *E*_ad_ value for this configuration (N2 site) is determined to be −2.18 eV, indicating stronger adsorption than the S-site interaction but weaker than the N1-site interaction. Charge-transfer analysis reveals that −0.113 e is shifted from the MB molecule to the adsorbent. To confirm the feasibility of the adsorption process, we also calculated the Gibbs' free energy change (Δ*G*) from the frequency calculations for all the complexes by including the entropy effect and ZPTE corrections. The Δ*G* values, given in Table 1, are all negative, confirming the exothermic nature and feasibility of the adsorption process under normal conditions.^66,67^

**Table 1:** Calculated bond distance (Å), adsorption energy (*E*_ad_ eV), dispersion-corrected adsorption energy (*E*_ad–D3_ eV), charge transfer (Q_CT_ e), Gibbs free energy change (Δ*G* eV), electron density (*ρ*(r) a.u), Laplacian of the electron density (∇^2^*ρ*(r) a.u), and energy density (*H*(r) a.u.) for MB molecule adsorption over the Al@C_24_N_24_ complex

Complex	X–Al (X = *N*, *S*)	*E*_ad_	*E*_ad–D3_	Δ*G*	*Q*_CT_	*ρ*(*r*)	∇^2^*ρ*(*r*)	*H*(*r*)
MB-Al@C_24_N_24_-N(1)	1.89, 2.50, 2.50	−3.07	−2.97	−2.43	−0.109	0.076 (Al–N), 0.012 (H⋯N)	0.445 (Al–N), 0.038 (H⋯N)	−0.0045, 0.0011
MB-Al@C_24_N_24_-S	2.37	−2.13	−2.03	−1.83	−0.149	0.049 (Al–S)	0.129 (Al–S)	−0.0012
MB-Al@C_24_N_24_-N(2)	2.00, 2.74, 2.52	−2.31	−2.18	−1.97	−0.113	0.061 (Al–N), 0.011 (H⋯N)	0.307 (Al–N), 0.036 (H⋯N)	−0.0040, 0.0011

**Fig. 3:**
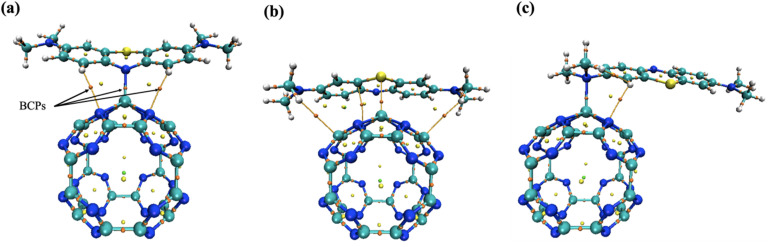
AIM molecular graphs of the MB-adsorbed Al@C_24_N_24_ complex involving the N(1) site (a), the S site (b), and the N(2) site (c). BCPs show bond critical points.

To gain deeper insights into the chemisorption mechanism of MB on the Al@C_24_N_24_ system, electronic structure analyses, including PDOS, CDD, and AIM were performed for the optimized adsorption configurations. The PDOS plots (Fig. 2) reveal a considerable overlap at the Fermi level between the p-orbitals of Al and the p-orbitals of N or S atoms of the dye. This overlap indicates the formation of a strong interaction and suggests chemisorption of the MB dye molecule over the surface of the Al@C_24_N_24_ system. The CDD plots for the adsorption of the MB molecule over the Al@C_24_N_24_ system (Fig. 2) demonstrate substantial electron-density redistribution at the interface for all configurations. This redistribution proves the strong interaction between the dye and the adsorbent surface, supporting the chemisorption mechanism. AIM analysis further confirms these findings, with the molecular graphs showing the three interactions of MB with Al@C_24_N_24_ visualized in Fig. 3. In the AIM analysis for configuration-1 (Fig. 3a), the values of *ρ*(*r*), ∇^2^*ρ*(*r*), and *H*(*r*) (Table 1) were calculated to be 0.076, 0.445, and −0.0045 a.u., respectively, for the N–Al bond. The high values of *ρ*(*r*) and ∇^2^*ρ*(*r*), along with the negative *H*(*r*) value, support the strong polar covalent interaction of the MB dye molecule with the surface of the Al@C_24_N_24_.^61,62^ The Al–S bond in adsorption configuration-2 (Fig. 3b) shows lower values of *ρ*(*r*) (0.049), ∇^2^*ρ*(*r*) (0.129), and *H*(*r*) (−0.012 a.u), signifying a moderate interaction with partial covalent character. Similarly, for adsorption configuration-3 (Fig. 3c), the N–Al bond exhibits *ρ*(*r*), ∇^2^*ρ*(*r*), and *H*(*r*) values of 0.06, 0.307, and −0.0040 a.u., respectively, implying a weaker electrostatic interaction with a covalent nature. Additionally, the idea of hydrogen bonds (N–H) being involved in configurations-1 and configurations-3, while van der Waals interactions being involved in configuration-2, has been suggested based on AIM analysis. Consequently, the AIM data provide strong evidence regarding the chemisorption of MB over the Al@C_24_N_24_ system, which results from the collective effects of covalent bonds, hydrogen bonds, and other intermolecular interactions.

**Fig. 4:**
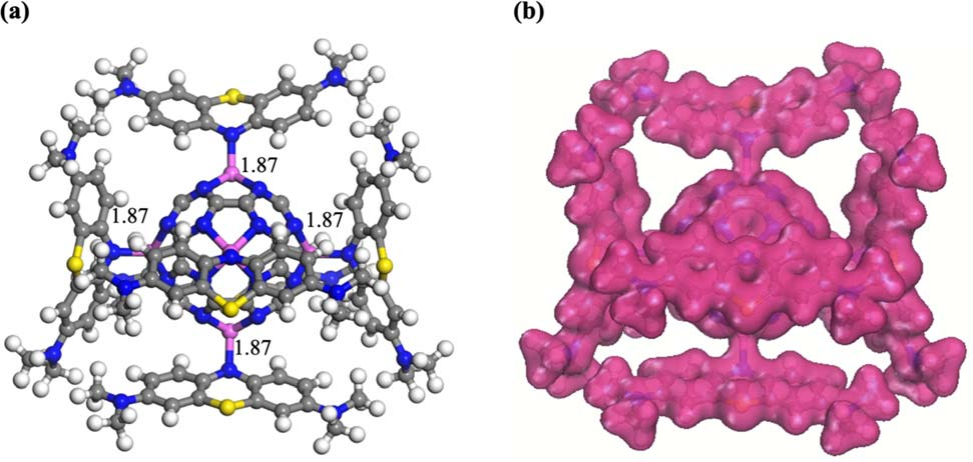
The optimized geometry of the 6MB-adsorbed Al_6_@C_24_N_24_ complex (a) and the corresponding charge density difference plot (b). All bond lengths are in Å.

To evaluate the maximum dye-uptake capability of the fullerene as an adsorbent, multiple MB molecules were interacted with the fully functionalized Al_6_@C_24_N_24_ fullerene and examined. The optimized geometry of six MB molecules simultaneously adsorbed over the surface of Al_6_@C_24_N_24_ in the form of a complex (represented as 6MB-Al_6_@C_24_N_24_) and the corresponding CDD plot, are displayed in Fig. 4a and b, respectively. As observed in the single MB adsorption study, the central (N1) atom of the MB molecule that was identified as the most feasible site of attachment was also considered for the interactions in the multi-adsorption configuration. After geometry relaxation, the MB molecules were found to adsorb strongly onto the Al_6_@C_24_N_24_ surface, with an average Al–N bond length of 1.87 Å in the 6MB-Al_6_@C_24_N_24_. This short bond distance indicates robust interactions between the dye and the adsorbent, signifying strong adsorption behavior. The adsorption energy per MB molecule for 6MB-Al_6_@C_24_N_24_ complex was found to be −3.05 eV. This highly negative value is indicative of chemisorption of the dye over the adsorbent surface. Hirshfeld charge analysis reveals an average charge transfer of −0.121 e from each MB molecule to the corresponding Al atom. This significant charge transfer facilitates the formation of strong Al–N1 covalent bonds, giving rise to effective MB adsorption over the Al_6_@C_24_N_24_ surface. The CDD plots further support this finding, showing substantial electron-density overlap between the MB molecules and the Al_6_@C_24_N_24_ system (Fig. 4b), which reinforces the chemisorption mechanism. These results demonstrate the exceptional adsorption capacity of the Al_6_@C_24_N_24_ system, accommodating multiple MB molecules without any structural distortion. This study highlights the potential applications of Al_6_@C_24_N_24_ as an efficient and robust adsorbent for dye removal applications.

To check the effect of solvation (hydration) on the adsorption of MB dye on Al@C_24_N_24_ adsorbent, the aqueous-phase solvation energy (*E*_solv_) was calculated, and the results were correlated with the gaseous-phase calculations. The aqueous-phase *E*_ad_ values and charge transfer (given in Table 2) were also investigated during MB interaction with the Al@C_24_N_24_ adsorbent to better understand the impact of solvation. Table 2 shows that the aqueous-phase *E*_ad_ values are lower (less negative) than the gas-phase adsorption values, yet they are still negative. The lower aqueous-phase *E*_ad_ values suggest that the MB adsorption over the Al@C_24_N_24_ adsorbent is weakened by the aqueous medium due to the solvation effect and the polar nature of MB. However, the thermodynamic feasibility and stabilization of the MB-Al@C_24_N_24_ complex are further supported by the negative *E*_solv_ values (Table 2), calculated for all three adsorption configurations of the MB dye over the adsorbent. Furthermore, as evident from the Hirshfeld charge analysis, charge transfer from MB to Al@C_24_N_24_ is also reduced in an aqueous environment relative to the gaseous phase. It is concluded that releasing the MB molecule from the Al@C_24_N_24_ adsorbent is energetically more favorable in aqueous medium relative to the gaseous phase, highlighting the potential effects of solubility on desorption in practical applications.

**Table 2:** Computed adsorption energy (*E*_ad_ eV) and (Q_CT_ e) for MB adsorption over the Al@C_24_N_24_ system in the aqueous phase

Complex	*E*_solv_	E_solv-D_3__	*E*_ad (aq)_	*E*_ad–D_3__	*Q*_CT_
MB-Al@C_24_N_24_-N(1)	−3.11	−2.88	−2.53	−2.44	−0.081
MB-Al@C_24_N_24_-S	−2.21	−2.13	−1.67	−1.52	−0.082
MB-Al@C_24_N_24_-N(2)	−2.37	−2.25	−1.81	−1.69	−0.069

**Fig. 5:**
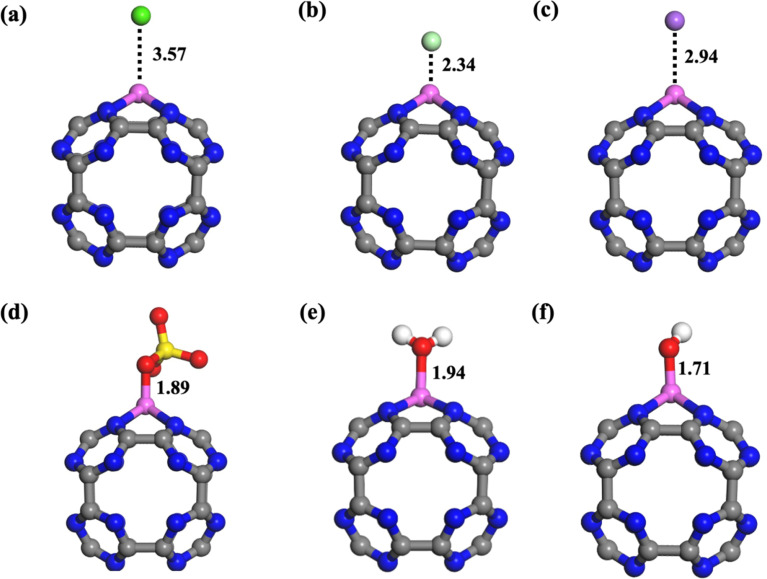
Optimized geometries of Ca^2+^ (a), Cl^−^ (b), Na^+^ (c), SO_4_^2−^ (d), H_2_O (e), and OH^−^ (f) adsorption over the Al@C_24_N_24_ adsorbent. All bond lengths are in Å.

To evaluate the selectivity of the Al@C_24_N_24_ adsorbent, the interaction of MB with the adsorbent in the presence of competitive ions, including OH^−^, Na^+^, Ca^2+^, Cl^−^, SO_4_^2−^, and H_2_O molecules, was simulated. The optimized geometries of these interacting species, together with the adsorbent, are displayed in Fig. 5, and their binding energies are tabulated in Table S4. The binding energies obtained for these competitive ions, except OH^−^, suggest that their presence would not hinder the MB adsorption over the Al@C_24_N_24_ adsorbent. Conversely, the OH^−^ ion exhibited strong binding (*E*_ad_ = −5.05 eV), probably due to its high charge density and strong basicity, allowing it to form a robust coordination with the electron-deficient Al site and likely obstructing MB access. Thus, it is inferred that the decontamination of MB from wastewater should be performed under neutral conditions, where the undesirable interaction is avoided due to the minimal OH^−^ ion concentration. However, although OH^−^ can bind strongly to the Al site, its realistic interference in a slightly alkaline medium is reduced by the adsorption kinetics of MB, driven by π–π stacking, electrostatic attraction, and the potential Al–N coordination, owing to the larger molecular size and multidentate nature of MB relative to OH^−^. The specificity of Al@C_24_N_24_ toward the MB dye over the OH^−^ ion is confirmed by the coadsorption complex (Fig. S10), obtained by placing a MB molecule near the Al active site of the adsorbent, where the OH^−^ ion is already bonded, followed by geometry optimization. The optimized co-adsorption complex shows that the N site of the MB is attached to the Al active site by pushing the OH group a little outward. The adsorption energy calculated for this system is −1.98 eV, which indicates that the adsorbent can also adsorb MB in an alkaline medium. In addition, to further examine the selectivity of Al@C_24_N_24_ for MB, we examined the competitive adsorption of Rhodamine B (RB) dye over the adsorbent. The optimized geometry of adsorbed Rhodamine B dye over the adsorbent (RB-Al@C_24_N_24_) is illustrated in Fig. S11. The intermolecular bonding parameters and adsorption energy (Table S4) clearly indicate that the RB adsorption over Al@C_24_N_24_ is weak compared to MB. This proves that Al@C_24_N_24_ exhibits high selectivity toward MB over RB and other common ions in aqueous environments, confirming its potential application for practical wastewater treatment (Table 3 and S4).

**Table 3:** Comparative analysis of MB adsorption over different adsorbents

Adsorbent	Capacity (mg g^−1^)	No. of MB adsorbed	E_ad_ (eV)	Computational/experimental	ref.
Graphene oxide	—	1	−2.25	Computational and experimental	68
Cd or ZnCd/TiO_2_	—	1	−1.15 or −1.127	Computational and experimental	69
Brazilian berries seed	188.26	1	−1.78	Computational and experimental	21
Alginic acid	51.34	1	−0.27	Computational and experimental	23
ZnTiO_3_(101)	—	1	−1.31	Computational	24
TiO_2_ (101)	—	1	−0.12	Computational	24
Polysaccharide-based composite hydrogel	122.1	—	—	Experimental	70
Hydrolyzed polyacrylamide	37.12	—	—	Experimental	71
*Tectona grandis* sawdust	172.41	—	—	Experimental	72
Cotton stalk	222.22	—	—	Experimental	73
Cellulosic olive stones biomass	88.2	—	—	Experimental	74
Walnut shell-carbon	315.00	—	—	Experimental	75
Oil palm shell-carbon	243.90	—	—	Experimental	76
Rice husk activated carbon	312	—	—	Experimental	77
Co/Ni-borophene	—	1	−2.99, −2.27	Computational	
G/C/dicyclohexyl, G/C/dimethylaminopropyl hydrochloride groups	274, 320	—	—	Experimental	78
TNTs/LDHs/OS	357	—	—	Experimental	79
SA/CMC-K composite microbeads	84.63	—	—	Experimental	80
Al_6_@C_24_N_24_	a2441	6	−3.05	Computational	This study

a Theoretical capacity (mg g^−1^).

**Fig. 6:**
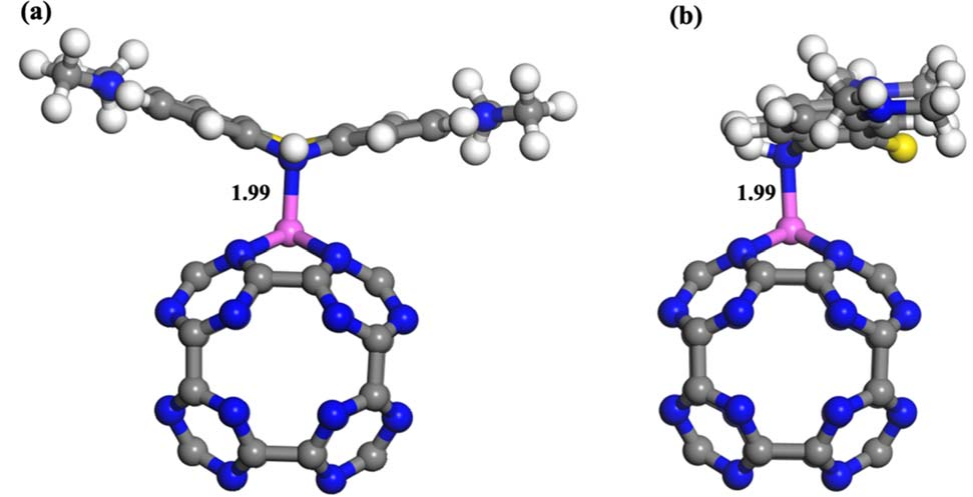
Relaxed structures of the LMB adsorption over the Al@C_24_N_24_ from the front view (a) and the side view (b). All bond lengths are in Å.

In an acidic environment, MB can convert into its reduced form, leuco-methylene blue (LMB),^81^ as depicted in Fig. 6. To investigate the adsorption characteristics of MB in such a medium, we examined the adsorption of LMB on the Al@C_24_N_24_ adsorbent. The optimized structure of LMB on Al@C_24_N_24_ is illustrated in Fig. 6. LMB is firmly adsorbed onto the Al-decorated C_24_N_24_ with a binding distance of 1.99 Å. The adsorption energy value for this complex is −2.06 eV, which indicates that the Al-decorated C_24_N_24_ system can effectively adsorb dye molecules in an acidic medium.

To assess the stability and experimental feasibility of the 6MB-Al_6_@C_24_N_24_ complex in a bulk aquatic environment, we applied the Amorphous Cell through Monte Carlo simulations with Adsorption Locator and Forcite code,^82^ implemented in the Material Studio Package. For this purpose, the DFT-optimized 6MB-Al_6_@C_24_N_24_ complex was initially enclosed inside a shell of around 80 water molecules in the amorphous cell, mimicking the bulk water system. The complex was initially optimized *via* the Amorphous Cell code, followed by full optimization through Forcite code using the COMPASS-III forcefield.^82^ During this optimization, 30 000 steps were set in the program, and the method of steepest descent was set in the minimizer.

**Fig. 7:**
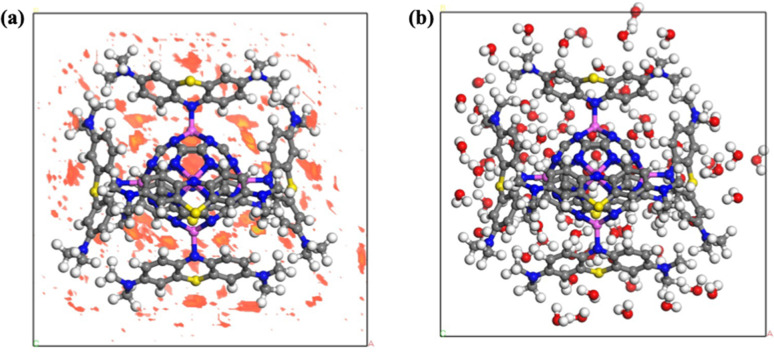
Representation of possible water interaction sites (a) and the optimized geometry of the 6MB-Al_6_@C_24_N_24_ in the amorphous cell containing 80 water molecules (b).

The red spots in Fig. 7a, being observed in the cell through Adsorption Locator analysis, predict the most probable interactive sites for water molecules with the complex. These interactive sites are exactly confirmed by the Forcite optimization (Fig. 7b), where water molecules can be seen around the complex at the positions of the red spots. The relaxed geometry in Fig. 7b shows that no distortion or bond dangling is observed and that the intermolecular bond distances are consistent with those obtained from DFT analysis, which reflects the stability of the complex in the aqueous environment. However, the stability of a singly adsorbed MB molecule over the adsorbent (MB-Al@C_24_N_24_) has been predicted by the negative *E*_solv_ value (Table 2). The stable existence of the complex in water further validates that the adsorbent can easily adsorb the dye molecules in an aquatic environment. Furthermore, these findings suggest that the results of the Forcite analysis are in close agreement with those of the Adsorption Locator analysis. The total electronic energy obtained *via* the COMPASS-III forcefield for the bulk structure is −5211.24 kcal mol^−1^. The negative electronic energy value suggests the thermodynamically stable nature of the complex in bulk water.

**Fig. 8:**
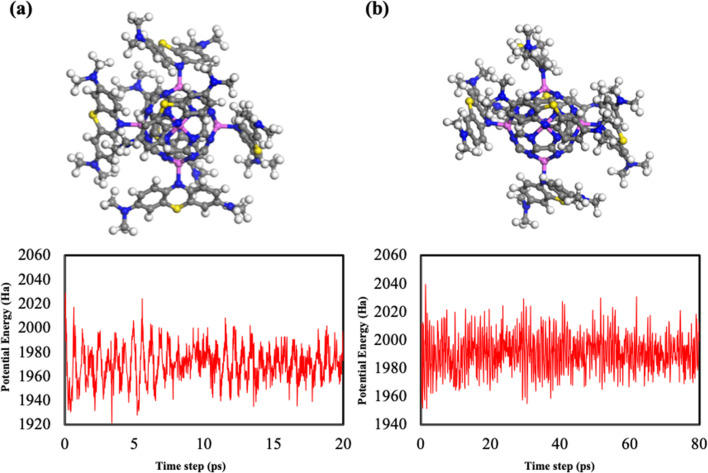
Molecular structure of the 6MB-Al_6_@C_24_N_24_ complex and its corresponding potential energy fluctuation plots after molecular dynamics simulations at 300 K for 20 ps with 1 fs (a) and 80 ps with 2 fs (b).

To further check the thermal stability of the 6MB-Al_6_@C_24_N_24_ complex in the bulk aquatic environment, we performed AIMD simulations *via* the Forcite code of the Amorphous Cell, with the complex surrounded by 80 water molecules, for a time period of 20 and 80 ps, with 1 and 2 fs at 300 K, respectively. The geometry after AIMD analysis and the potential energy fluctuation plots are presented in Fig. 8a and b; water molecules are excluded for simplicity to clearly visualize the intermolecular bonding sites between the dye and the adsorbent. The simulations show that the complex is highly stable (negligible intermolecular bond variations are observed) in the bulk environment; even when the temperature of the water is increased, the adsorbent can still stably adsorb the dye molecules from water.

The interaction of the adsorbate and adsorbent is of utmost significance in the adsorption phenomenon. Nonetheless, the recovery of adsorbents *via* desorption of adsorbates is the essence of an effective adsorption, which cannot be overlooked. As mentioned, the *E*_ad_ values for MB adsorption over Al@C_24_N_24_ by the three configurations (Fig. 2) were −2.97, −2.18, and −2.08 eV, respectively. These values reflect the irreversible adsorption of MB molecules over the surface of Al@C_24_N_24_, where the desorption does not occur unless special conditions are applied. At higher temperatures, the bond between MB and the adsorbent surface is likely to weaken due to entropic effects, which generally facilitates the recyclability of the adsorbate. To quantitatively assess this, the desorption time (*τ*) of the adsorbed MB was calculated using the following relation:^83^3
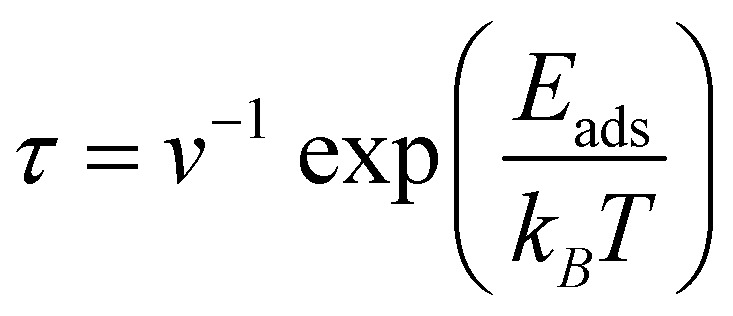
In this context, *T* denotes the ambient temperature, which is 298.15, 398.15 and 498.15 K, while *k*_*B*_ is Boltzmann's constant, and *v* is the attempt frequency, set at 10^12^ Hz. This formula indicates that the desorption time is exponentially related to *E*_ad_, suggesting that even slight changes in binding strength can result in significant variations in desorption behavior. The predicted *τ* values (Table S5) for MB desorption from the Al@C_24_N_24_ surface are 1.63 × 10^38^, 2.03 × 10^21^, and 7.40 × 10^24^ s at 298.15 K, which decrease considerably as the temperature increases. It is worth mentioning that the calculated *τ* values correspond to idealized gas-phase interactions, obtained from DFT calculations. As observed from the solvent effect, the adsorption energies are observed to decrease (less negative) in aqueous medium. The decrease in interactive forces of the adsorbate–adsorbent complex is probably due to solvation effects, competitive water molecules' interactions with the adsorbent, and entropic contributions, which facilitate desorption by reducing the desorption barrier. Thus, it has been suggested that increasing the temperature of the aqueous medium would accelerate desorption of MB from the adsorbent in practice, ensuring regeneration of the adsorbent. Therefore, the adsorbent surface not only offers selective and stable adsorption of MB molecules but also facilitates their efficient desorption, a feature that is highly advantageous for practical pollutant sensing applications.

## 4 Conclusion

In this study, the potential applications of the Al@C_24_N_24_ complex as an adsorbent for scavenging carcinogenic MB dye from wastewater were investigated by DFT calculations. The results reveal that Al atoms strongly adhere to the C_24_N_24_ fullerene with high *E*_ad_ values of −6.91 and −5.18 eV at the DFT-D3 level, with the incorporation of single and multiple (six) Al atoms, respectively. Electronic properties analysis underscores Al sites on the C_24_N_24_ fullerene as the primary reactive sites for the adsorption of MB molecules. Similarly, structural information and AIM analysis indicate that MB is physisorbed over the pristine C_24_N_24_ system with an *E*_ad_ value of −0.49 eV. In contrast, MB molecules are chemisorbed over the Al@C_24_N_24_ surface *via* N1, S, and N2 sites, with significantly shorter bond lengths (*e.g.*, 1.89, 2.37, and 2.00 Å). These observed shorter distances are indicative of strong chemisorption, accompanied by higher *E*_ad_ values of −2.97, −2.08, and −2.18 eV at the DFT-D3 level. Moreover, the CDD, PDOS, and AIM analyses confirm that the interaction between the MB molecule and the Al@C_24_N_24_ surface is governed by covalent and hydrogen bonding. The adsorption capacity assessment reveals that Al-decorated C_24_N_24_ can accommodate up to six MB molecules, highlighting its high dye-uptake potential. Additionally, the solvation effect is deduced to facilitate the desorption of MB molecules from the surface of the Al@C_24_N_24_. To further check the solvation effect, we also performed simulations for the 6MB-Al_6_@C_24_N_24_ complex in an aqueous environment by placing the complex in an amorphous cell with 80 water molecules. The geometry optimization and thermal stability study at 300 K show that the complex is highly stable in aqueous medium. This study represents the first demonstration of how Al incorporation significantly enhances the performance of C_24_N_24_ fullerene for MB removal from wastewater, providing insights into developing effective carcinogen scavengers for water purification.

## Author contributions

Habib Ullah: conceptualization, software, validation and writing – original draft. Zakir Zaman Khan: validation, writing, review and editing. Akif Safeen: formal analysis and editing original draft. Adnan Ali Khan: computational tools, writing, review and editing. Noor Ul Islam: data curation, review and editing. Ghafar Ali: investigation, writing, review & editing. Basit Ali: funding acquisition, writing, review & editing the manuscript with helpful input and insight. Imran Shakir: formal analysis, review and editing. Xie Yi: supervision, project administration, writing, review and editing.

## Conflicts of interest

The authors declare that they have no known competing financial interests or personal relationships that could have appeared to influence the work reported in this paper.

## Supplementary Material

RA-016-D6RA00172F-s001

## Data Availability

The data supporting this article have been included as part of the supplementary information file (SI). Supplementary information is available. See DOI: https://doi.org/10.1039/d6ra00172f.
