# Supplementary material for: Design of Al-decorated C24N24 fullerene for efficient adsorption and removal of methylene blue dye from water
Source: RSC Adv. 2026 Feb 19;16(11):9709–21. doi: 10.1039/d6ra00172f (PMC12917590; doi:10.1039/d6ra00172f)
Supplement: RA-016-D6RA00172F-s001 [file RA-016-D6RA00172F-s001.pdf]

Supplementary Information (SI) for RSC Advances.
This journal is © The Royal Society of Chemistry 2026

Supplementary Materials

Design of Al decorated $C_{24}N_{24}$ fullerene for efficient adsorption and removal of methylene blue dye from water

Habib Ullah[a], Zakir Zaman Khan[b], Akif Safeen[c], Adnan Ali Khan[d], Noor Ul Islam[e], Ghafar Ali[f], Basit Ali*[g], Imran Shakir[h], and Yi Xie*[a]

[a]State Key Laboratory of Silicate Materials for Architectures, Wuhan University of Technology, Wuhan 430070, P.R. China

[b]Department of Chemistry, University of Malakand, Chakdara, Dir Lower 18800, Khyber Pakhtunkhwa, Pakistan

[c]Department of Physics, University of Poonch Rawalakot, Rawalakot, 12350, Pakistan

[d]School of Science, Harbin Institute of Technology (Shenzhen), Shenzhen, China

[e]Government Degree Collage Lalqilla Dir Lower, Lalqilla 18350, Khyber Pakhtunkhwa, Pakistan; and Higher Education Department Khyber Pakhtunkhwa, Peshawar, Pakistan

[f]Nanomaterials Research Group (NRG), Physics Division, PINSTECH, Nilore, Islamabad, Pakistan

[g]Department of Chemistry and Materials Science, Aalto University, P.O. Box 16100, FI-00076 Aalto, Finland

[h]Department of Physics, Faculty of Science, Islamic University of Madinah, Madinah 42351,

Saudi Arabia

*Correspondence authors

basit.ali@aalto.fi (Basit Ali)
xiey@whut.edu.cn (Yi Xie)

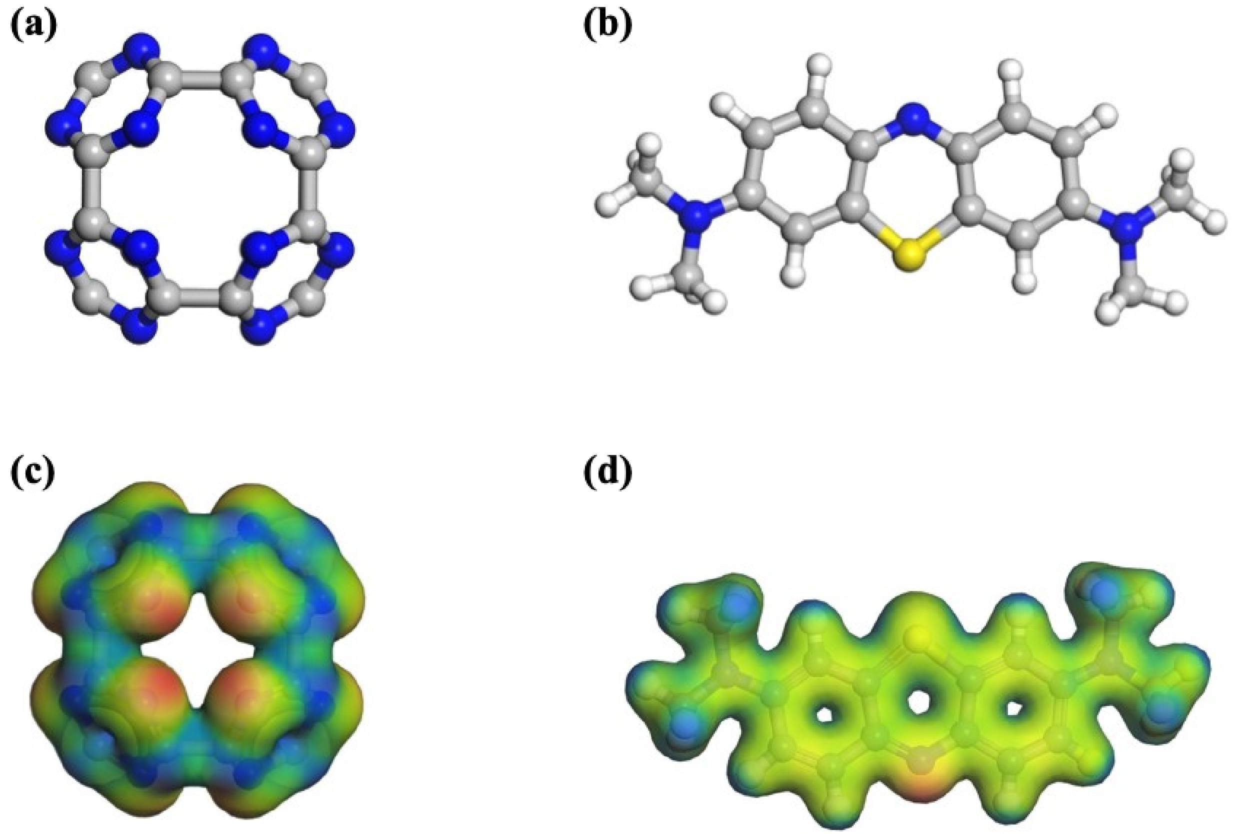

Fig. S1. Optimized geometry of pristine $C_{24}N_{24}$ fullerene (**a**), MB dye (**b**), and their corresponding molecular-electrostatic potential map (**c, d**).

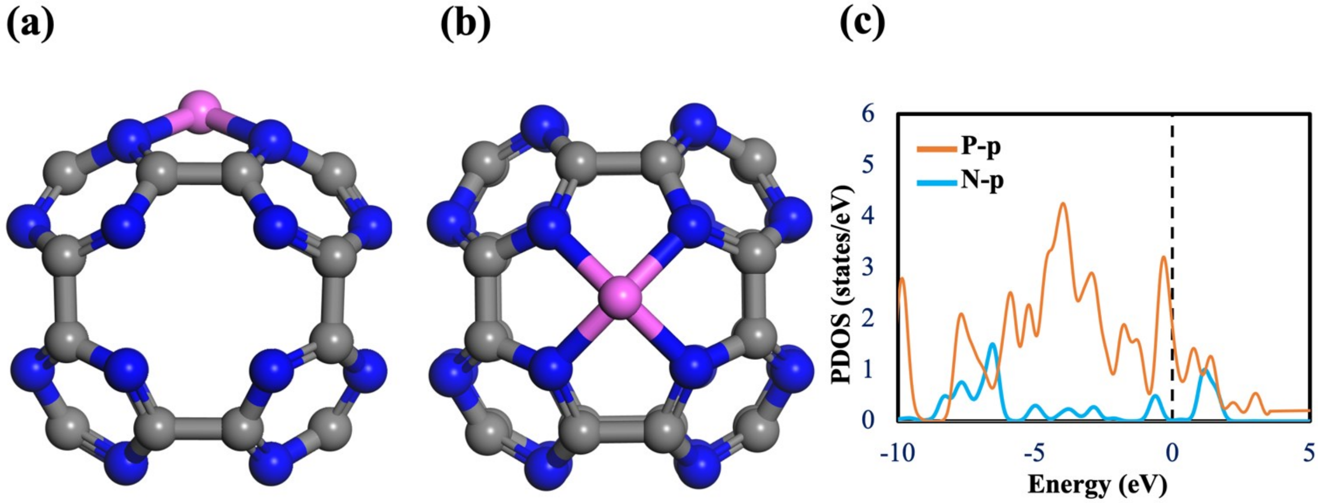

Fig. S2. Optimized geometry of the single Al incorporated $C_{24}N_{24}$ fullerene (Al@$C_{24}N_{24}$) from the top view (**a**) and front view (**b**), along with the corresponding partial density of state plot (**c**).

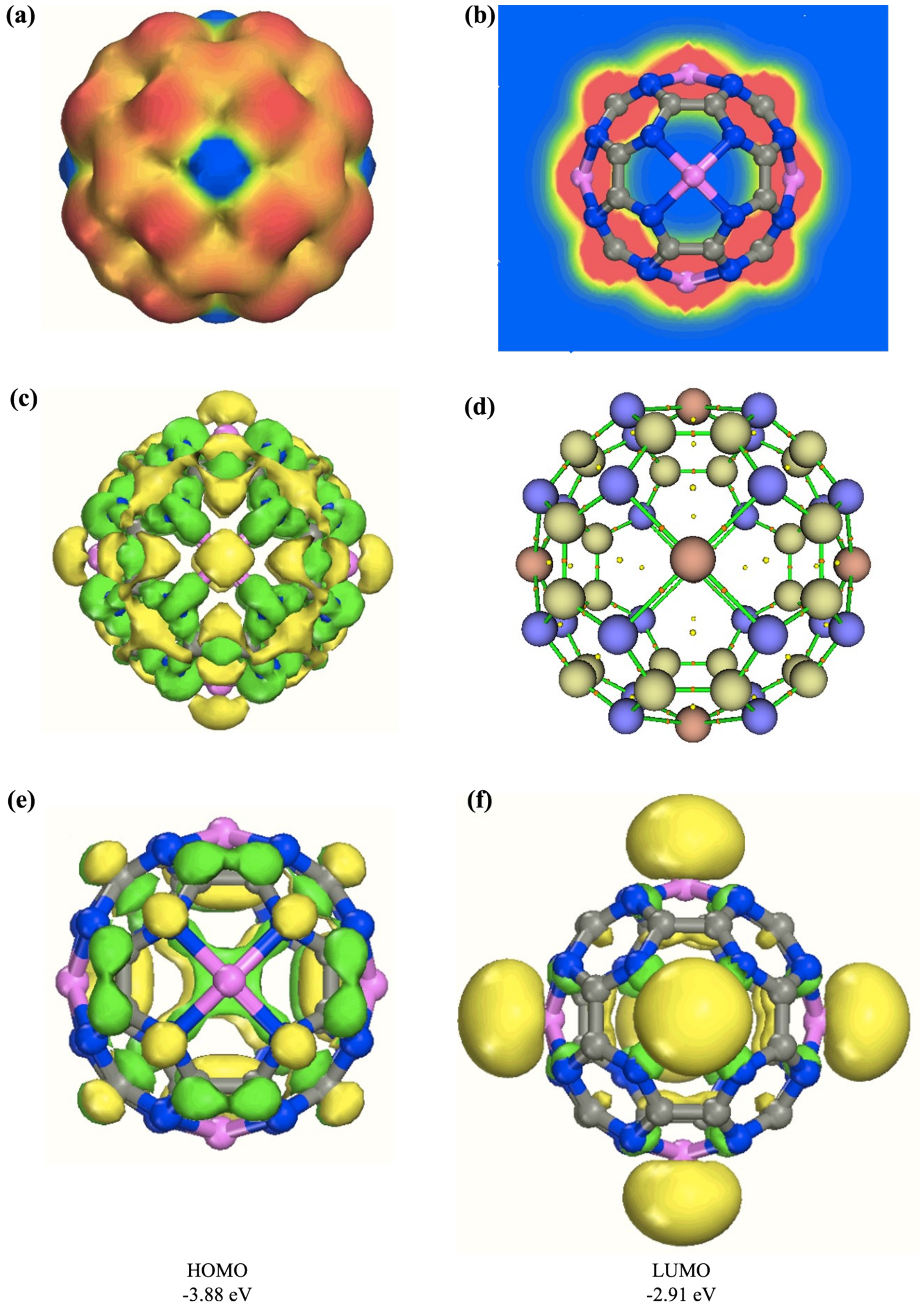
(a)
(b)
(c)
(d)
(e)
(f)
HOMO
-3.88 eV
LUMO
-2.91 eV

Fig. S3. Molecular-electrostatic potential map (**a**), charge-density difference contour (**b**), deformation-charge density map (**c**), AIM molecular graph (**d**), and HOMO-LUMO (**e**, **f**) of $Al_6@C_{24}N_{24}$ system

(a) **(b)**

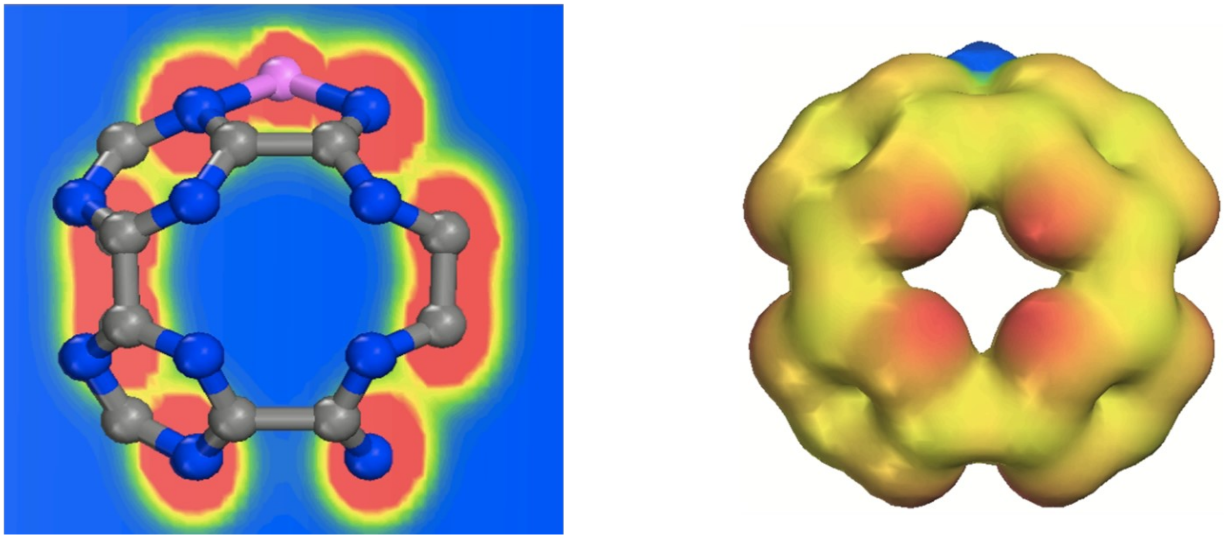

Fig. S4. Charge-density difference (**a**) and molecular-electrostatic potential map (**b**) plot for $Al@C_{24}N_{24}$ fullerene

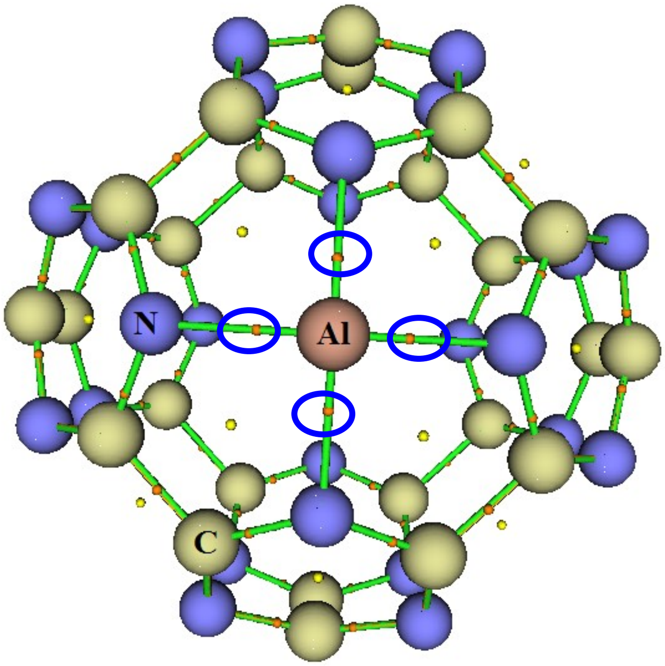

Fig. S5. Graphical representation of AIM plot of the $Al@C_{24}N_{24}$ fullerene. The orange color dots encircled with blue dotted line show the bond critical points between Al–N bond

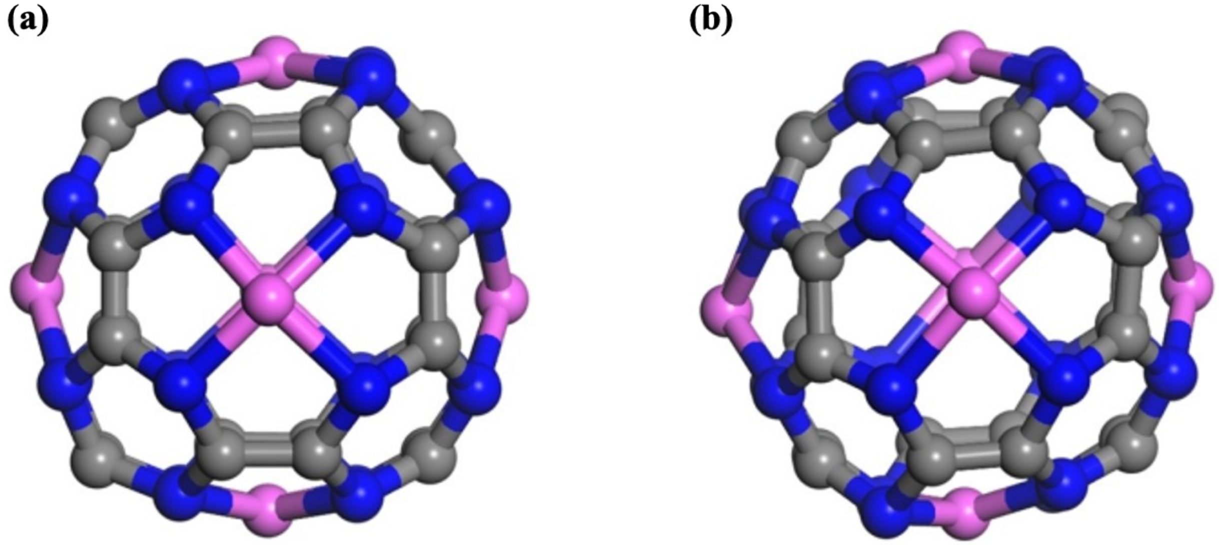

Fig. S6. Structure of $Al_6@C_{24}N_{24}$ after AIMD simulations performed at 500 K (**a**) and 1000 K (**b**) for 2 ps

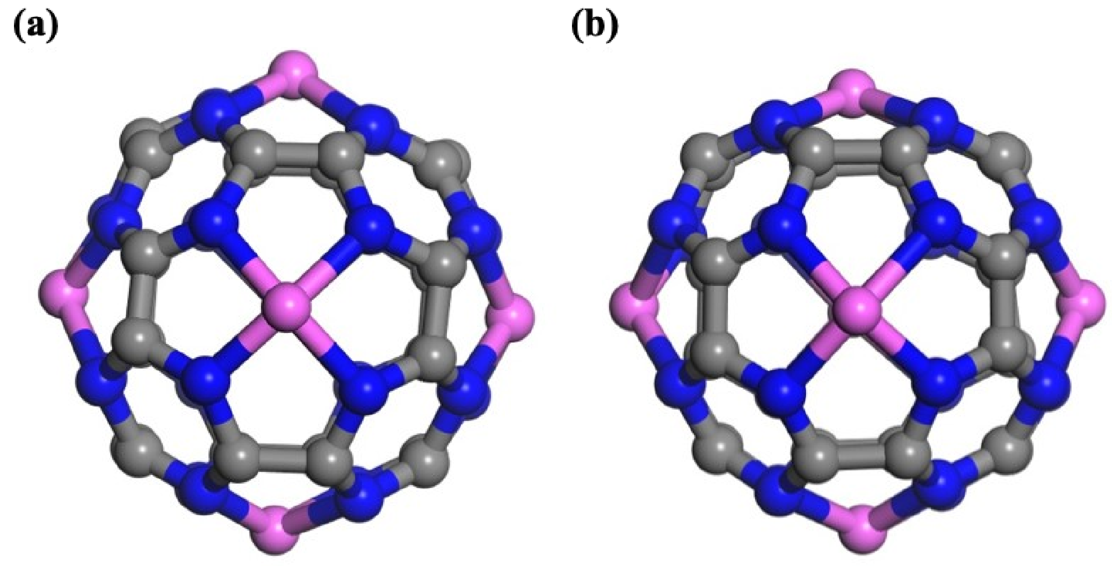

Fig. S7. Structure of $Al_6@C_{24}N_{24}$ after AIMD simulations performed at 500 K (**a**) and 1000 K (**b**) for 10 ps

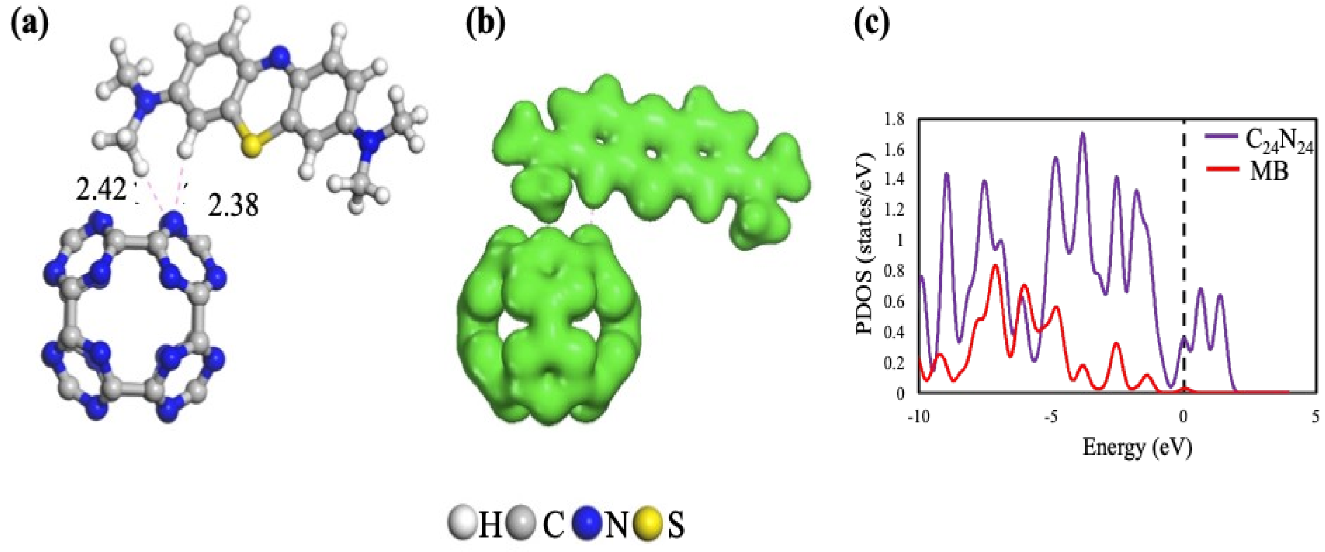

Fig. S8. Optimized geometry of $C_{24}N_{24}$ nanocage with adsorbed MB dye (**a**), corresponding Charge-density difference map (**b**) and partial density of state plot (**c**). All distances are in Å

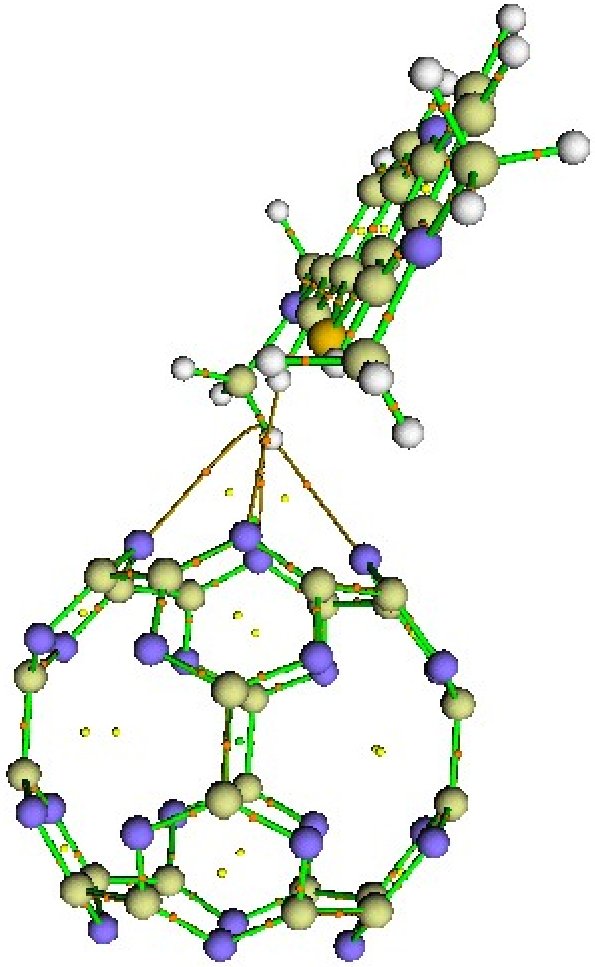

Fig. S9. AIM plot for MB adsorption over bare $C_{24}N_{24}$ fullerene. Golden color line and ball represent the bond critical points

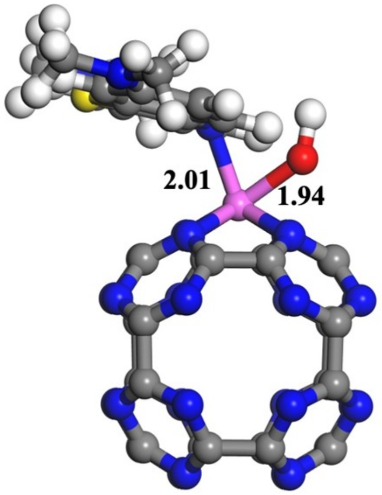

Fig. S10. Optimized geometry of the MB adsorption over the OH-Al@$C_{24}N_{24}$ system. All distances are in Å

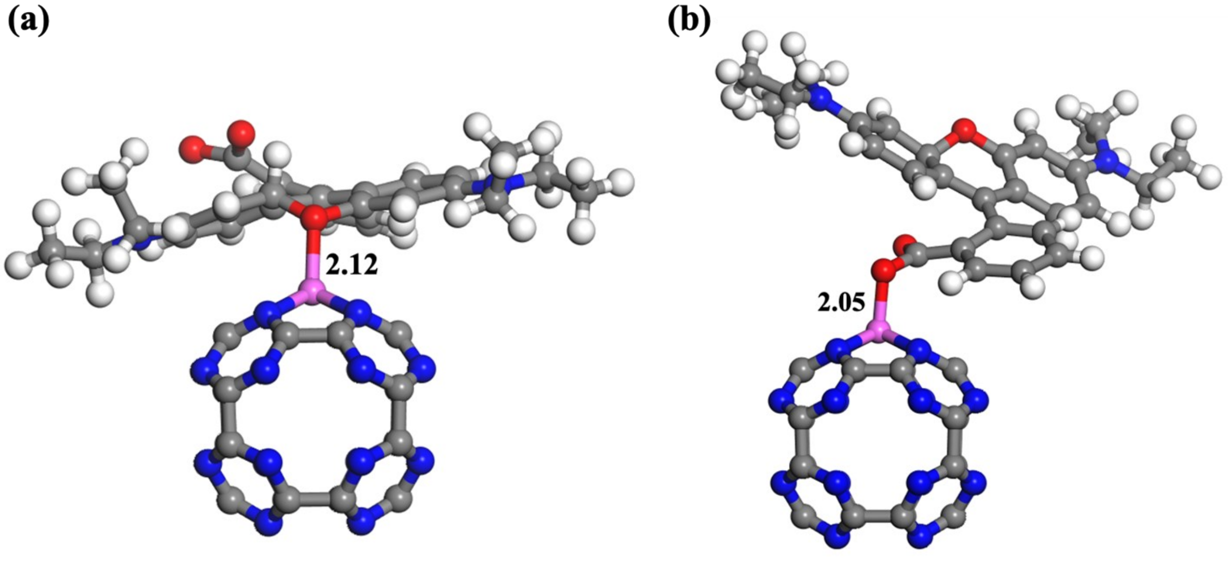

Fig. S11. Relaxed structure of Rhodamine B dye adsorbed over Al@$C_{24}N_{24}$ through Xanthene oxygen (**a**) and carboxyl oxygen (**b**). All distances are in Å

Table S1. The computed electron density (ρ(r) a.u.), its Laplacian ($\nabla^2\rho$(r) a.u.) and total electron energy density (H(r) a.u.) (au) for N–Al bonds in Al@$C_{24}N_{24}$ system

Bond	ρ(r)	$\nabla^2\rho$(r)	H(r)
N–Al (1)	0.072	0.450	–0.0017
N–Al (2)	0.072	0.450	–0.0017
N–Al (3)	0.072	0.450	–0.0017
N–Al (4)	0.072	0.450	–0.0017

Table S2. The computed electron density (ρ(r) a.u.), its Laplacian ($\nabla^2\rho$(r) a.u.) and total electron energy density (H(r) a.u.) for N–Al bonds in Al_6@$C_{24}N_{24}$ system

Bond	ρ(r)	$\nabla^2\rho$(r)	H(r)
N–Al (1)	0.077	0.422	–0.0094
N–Al (2)	0.077	0.422	–0.0094
N–Al (3)	0.077	0.422	–0.0094
N–Al (4)	0.077	0.422	–0.0094

Table S3. Calculated intermolecular bond length (Å), adsorption energy (E_{ad} eV), charge density transfer (Q_{CT} e), electron density (ρ(r) a.u.), Laplacian of electron the density ($\nabla^2\rho$(r) a.u.), and energy density (H(r) a.u.) for adsorption of MB over the bare $C_{24}N_{24}$ nanocage

Complex	H---B	E_{ad}	Q_{CT}	ρ(r)	$\nabla^2\rho$(r)	H(r)
MB-$C_{24}N_{24}$	2.42, 2.38	–0.49	0.001, 0.004	0.008,	0.033	0.0017
				0.008	0.031	0.0012

Table S4. Comparative analysis of the adsorption of various competitive species over the Al@$C_{24}N_{24}$ adsorbent. R (Å) for intermolecular distance and E_{ad} (eV) for adsorption energy

System	R (Å)	E_{ad} (eV)
Ca-Al@$C_{24}N_{24}$	3.57	0.24
Cl-Al@$C_{24}N_{24}$	2.34	–0.31
Na-Al@$C_{2s4}N_{24}$	2.94	–0.11
SO_4-Al@$C_{24}N_{24}$	1.89	–2.51
H_2O-Al@$C_{24}N_{24}$	1.94	–1.54
OH-Al@$C_{24}N_{24}$	1.71	–5.05
RB-Al@$C_{24}N_{24}$	*2.12/♦2.05	*–1.89/♦–2.43

* Xanthene oxygen interaction of RB with the adsorbent

♦ Carboxyl Oxygen interaction of RB with the adsorbent

Table S5. Desorption time (τ) of MB from the Al@$C_{24}N_{24}$ surface at various temperature (K)

Complex	E_{ad-D3} (eV)	τ @ 298 K (s)	τ @ 398 K (s)	τ @ 498 K (s)
MB-Al@$C_{24}N_{24}$-N(1)	–2.97	1.63×10^{38}	3.95×10^{25}	1.13×10^{18}
MB-Al@$C_{24}N_{24}$-N(2)	–2.03	2.03×10^{21}	4.20×10^{13}	2.58×10^{8}
MB-Al@$C_{24}N_{24}$-S	–2.18	7.40×10^{24}	3.70×10^{15}	1.15×10^{10}
